# Bone loss in Gorham’s disease: A case study

**DOI:** 10.3892/etm.2013.940

**Published:** 2013-01-31

**Authors:** BIN CHEN, XIAOFENG LV, JINXIAO WU, XINGGUANG ZHANG, XIUMIN JIAO, JING ZHAO, QIANPENG CHENG, CAN CUI

**Affiliations:** 1 Department of Endocrinology and Metabolism, The Military General Hospital of Beijing PLA, Beijing 100700;; 2 Department of Endocrinology and Metabolism, The 2nd Affiliated Hospital, Harbin Medical University, Harbin 150080, P.R. China

**Keywords:** ribs, maxillary bone, Gorham’s disease, radiotherapy, prognosis

## Abstract

In this study, a rare disease with characteristics of spontaneous osteolysis of the bone is reported. The patient was an eight-year-old male, who was admitted with shortness of breath. The absence of the right clavicle was identified by radiography. However, the change to the right clavicle was not malignant, as indicated by bone scanography. The biopsy of the right cervical rib revealed a number of vascular fibrous tissues and vessels distending and shunting together; however, no cell proliferation was observed. In addition, no acid-fast bacillia or malignant cells were detected in the sample of pleural effusion from the patient. Low hemoglobin (93 g/l) and a slight elevation of alkaline phosphatase levels (133 U/l) were observed; however, the other laboratory examination results were normal. The follow-up investigation and radiotherapy results indicated that the osteolysis of the skull and the other portion of bone had not worsened. Although it has been reported that >15% of patients succumb to this disease, the patient reported in the current study was in a relatively stable condition.

## Introduction

Gorham’s disease (Gorham-Stout syndrome) is a rare disorder characterized by progressive resorption of the whole or part of a bone (1–5). It is non-hereditary and often occurs in individuals aged <40 years without gender-related differences (6). The pathogenetic mechanism of Gorham’s disease remains unknown. Phase I of this disease represents increased vascular concentration in the bone-displacing fibrous tissues. In phase II, only fibrous tissue is observed (7,8). There is controversy with regard to the presence or absence of osteoclasts in this condition. In certain cases, osteoclastic activity is minimal or non-existent, whereas in other cases, osteoclasts are easily identifiable (9). During the past 10 years, we have identified only 2 cases that were diagnosed as Gorham’s disease. In the current study, we report one of these cases.

## Case report

**Figure 1:**
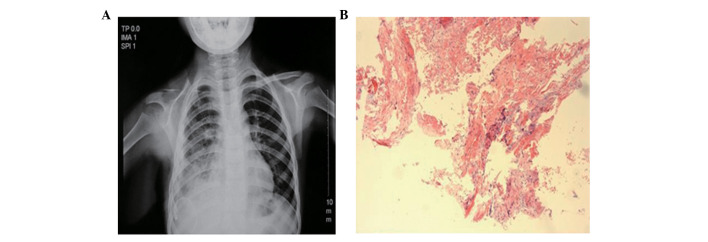
(A) An X-ray image of the thorax. The X-ray image revealed that the right clavicle was absent, the anterior extremities of the right ribs were destructed and pleural effusion was observed on the right side of the thorax. (B) Histological changes of the right cervical ribs. Three pieces of tissue were removed from the right cervical ribs. One was a sample of non-uniform tissue with a size of 0.8×0.4×0.2 cm, while the others were two pieces of grain-like gray tissue. Proliferation of fat and fibrous connective and vascular tissue were observed under an optical microscope. Parts of vessels distended and shunted together to form a construction similar to a sinus. Hyperplastic capillaries aggregated together with monolayer flat endothelium cells coating the wall of the lumen. Proliferative collagen, a few elastic fibers and smooth muscles were observed with Masson’s stain.

An eight-year-old male in West China was admitted with shortness of breath for 10 months. The patient was admitted to Department of Endocrinology and Metabolism, The Military General Hospital of Beijing PLA, Beijing, on September 13, 2010. Radiographs revealed the absence of the right clavicle, destruction of the anterior extremity of the right ribs and pleural effusion of the right side (Fig. 1A). Bone scanography indicated that the change in the right clavicle was not malignant. The biopsy of the right cervical rib revealed considerable vascular fibrous tissue with certain vessels distending and shunting together to form sinus construction (Fig. 1B), in which there was no endothelial cell proliferation. Masson’s staining revealed the proliferation of collagen.

Acid-fast bacillia and malignant cells were not detected in a sample of pleural effusion; however, the Rivalta and Chyle tests were positive. Other laboratory investigation results were normal, with the exception of low hemoglobin (93 g/l) and a slight elevation of the alkaline phosphatase levels (133 U/l). In the follow-up examination there was no progress of osteolysis of the skull and the other portion of bone. Prior written and informed consent were obtained from every patient and the study was approved by the ethics review board of The Military General Hospital of Beijing PLA.

## Discussion

One case of Gorham’s disease was diagnosed according to the clinical manifestations, radiological images and the changes of histopathology. There was no family history of bone disease or trauma. The portions of bone loss were on the rib cage and maxillary bone.

The treatment options utilized previously consist of surgical resection and radiotherapy and mainly target the patient’s symptoms (6,10–14). The disparity of the radio-sensitivity of Gorham’s disease-specific cells has resulted in mixed results following treatment (12–14). In patients with active osteoclasts, anti-resorptive therapy, including bisphosphonates or calcitonin, may improve the progressive osteolytic changes (15–17).

In the current study, radiotherapy was administered to the patient who was identified to have no osteoclasts when examined histologically. Despite the report that >15% of patients succumb due to this disease, the patient was well with no worsening of the condition. The prognosis depends on complications, including neurological deficits and pleural effusion (11,18). Life expectancy is not affected if the extremities are involved.
